# Structural Features and Biological Properties of Ellagitannins in Some Plant Families of the Order Myrtales

**DOI:** 10.3390/ijms11010079

**Published:** 2010-01-06

**Authors:** Takashi Yoshida, Yoshiaki Amakura, Morio Yoshimura

**Affiliations:** College of Pharmaceutical Sciences, Matsuyama University, Bunkyo-cho, Matsuyama, Ehime 790-8578, Japan; E-Mails: amakura@cc.matsuyama-u.ac.jp (Y.A.); myoshimu@cc.matsuyama-u.ac.jp (M.Y.)

**Keywords:** ellagitannins, *C*-glycosidic ellagitannins, oligomeric ellagitannins, Myrtales, biological activity

## Abstract

Plant tannins, including hydrolysable and condensed varieties, are well known antioxidants in medicinal plants, foods, and edible fruits. Their diverse biological properties and potential for disease prevention have been demonstrated by various *in vitro* and *in vivo* assays. A number of ellagitannins, the largest group of hydrolysable tannins, have been isolated from dicotyledoneous angiosperms and characterized. This diverse class of tannins is sub-grouped into simple ellagitannins, *C*-glycosidic ellagitannins, complex tannins (condensates of *C*-glycosidic tannins with flavan-3-ol), and oligomers up to pentamers. This review outlines and describes the chemotaxonomic significance of structural features in various types of ellagitannins found in plants belonging to the Myrtaceae, Onagraceae, and Melastomataceae families, which are all included in the order Myrtales. Any biological activities that have been reported, including antitumor and antibacterial effects as well as enzyme inhibition, are also reviewed.

## 1. Introduction

Plant tannins, one of the major groups of antioxidant polyphenols found in food and beverages, have attracted a lot of attention in recent years because of their multifunctional properties beneficial to human health. These diverse tannins may be divided into two large groups: condensed and hydrolysable. Condensed tannins are formed through the condensation of flavan-3-ols (catechins) and are often referred to as proanthocyanidins. Among the more than 500 hydrolysable tannins hitherto characterized, ellagitannins, which produce ellagic acid upon hydrolysis, constitute the largest group; the remaining group is gallotannins (galloylglucoses). The ellagitannins include: (1) monomeric ellagitannins, (2) *C*-glycosidic ellagitannins with an open-chain glucose core, (3) condensates of *C*-glycosidic tannins with flavan-3-ol (complex tannin), and (4) oligomers which are produced through intermolecular C-O or C-C bonds between monomers [1,2]. Unlike the condensed tannins that are widespread throughout the plant kingdom, ellagitannins have been found only in dicotyledoneous angiosperms. Among the plant families rich in ellagitannins are the Myrtaceae, Lythraceae, Onagraceae, Melastomataceae, and Combretaceae [3]. These families belong to the order Myrtales according to the plant classification systems of New Engler, Cronquist, and APGII (angiosperm phylogeny group) [4]. Ellagitannins have also been isolated from plant species of Trapaceae and Punicaceae, which belong to Myrtales in Cronquist’s and the New Engler’s systems. This review outlines and describes the chemotaxonomic significance of structural features found in various types of ellagitannins, focusing on representative examples found in the plants of Myrtales. Any observed antioxidative and antitumor effects of these ellagitannins are also reviewed.

## 2. Monomeric Ellagitannins

### 2.1. Simple Ellagitannins

**Figure 1.**
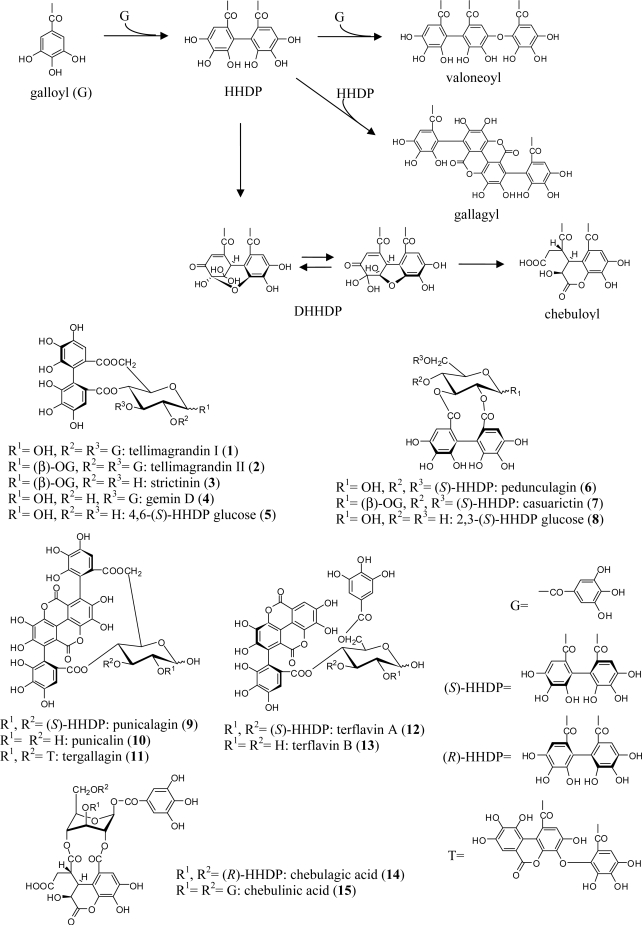
Structures of monomeric ellagitannins **1**–**15**.

Ellagitannins are characterized by the presence of one or more hexahydroxydiphenoyl (HHDP) unit(s) on a glucopyranose core. The HHDP group is biosynthetically formed through intramolecular, oxidative C-C bond formation between neighboring galloyl groups in galloylglucoses [5]. They are easily hydrolysed, either enzymatically or with acid, to liberate a stable ellagic acid as the dilactone form of hexahydroxydiphenic acid. In addition to the HHDP group, other constituent acyl units in ellagitannins include a galloyl group and HHDP metabolites such as valoneoyl, dehydrohexahydroxydiphenoyl (DHHDP), and chebuloyl groups. Variations in the number and position of these acyl units on the glucose core provide a variety of analogs such as tellimagrandin I (**1**), and II (**2**), pedunculagin (**6**), casuarictin (**7**) [6], chebulagic acid (**14**), and chebulinic acid (**15**) [7] (Figure 1). Note that the chiral HHDP group at O-2/O-3 and O-4/O-6 of the glucose residue has an *S*-configuration, whereas that at O-3/O-6 has an *R*-configuration, as indicated by a positive and negative Cotton effect around 230 nm in their respective circular dichroism (CD) spectra [8].

Representative ellagitannin monomers thus far isolated from the Myrtaceae, Melastomataceae, Onagraceae, Trapaceae, Combretaceae, and Punicaceae families are summarized in Figure 1 and Table 1.

Ellagitannins having a ^4^C_1_-glucopyranose core, e.g., **1**–**8**, have been isolated along with gallotannins from various other plant families and show little chemotaxonomic significance. The considerably rare tannins punicalagin (**9**) and punicalin (**10**), both of which contain a gallagyl unit and were first isolated from the pericarps of pomegranate (*Punica granatum* Punicaceae) [40], were a characteristic component in some *Terminalia* species. This supports the hypothesis that Punicaceae is chemotaxonomically proximate to Combretaceae as classified by Cronquist and Engler. The punicalagin analogs tergallagin (**11**) and terflavins A (**12**) and B (**13**) were also isolated from *T. chebula* [35] and *T. catappa* [34].

Some *Terminalia* species produce ellagitannins with a ^1^C_4_-glucopyranose core and a unique chebuloyl group, such as chebulagic acid (**14**) and chebulinic acid (**15**). Although **14** and **15** have also been found in plants of the *Geranium* [41] and *Euphorbia* genera [42], their co-occurrence with **9** and/or **10** is a chemotaxonomic feature of *Terminalia*.

### 2.2. C-Glycosidic Ellagitannins

**Figure 2.**
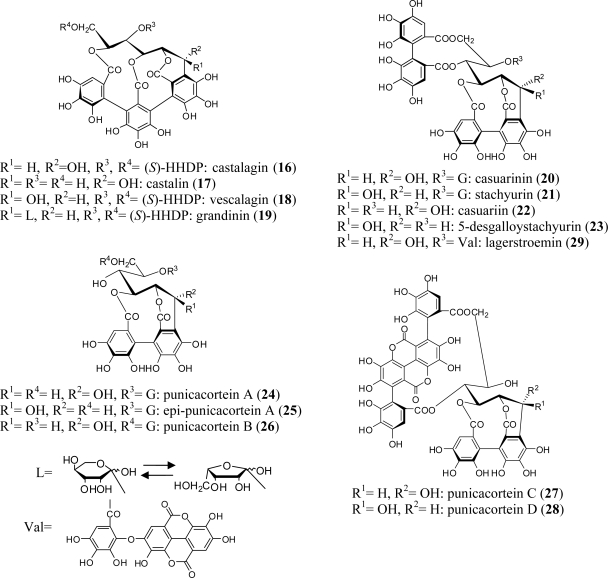
Structures of *C*-glycosidic ellagitannins **16**–**29**.

*C*-Glycosidic ellagitannins have been found in many plant families, including Lythraceae, Myrtaceae, Combretaceae, Melastomataceae, and Punicaceae, as well as Fagaceae, Betulaceae, Casuarinaceae, Rosaceae, Theaceae, and Elaeagnaceae [1]. They are categorized into two types: castalagin-type, which contain a flavogalloyl unit participating in the *C*-glucosidic linkage, such as castalagin (**16**) and its C-1 epimer, vescalagin (**18**), and casuarinin-type, which contain an HHDP unit, such as casuarinin (**20**) and stachyurin (**21**). In addition to these tannins, their metabolites, *i.e.*, grandinin (**19**), casuariin (**22**), and 5-desgalloylstachyurin (**23**), have been isolated from various plants of the Myrtales (Figure 2). *Lagerstroemia flos-reginea* and *L. speciosa* (Banaba) belonging to the family Lythraceae are particularly rich in *C*-glycosidic tannins including **16**, **18**, and **20**–**23** and casuarinin-type metabolites including punicacortein A (**24**) and lagerstroemin (**29**). Punicacortein A (**24**) and its analogs epi-punicacortein A (**25**) and punicacorteins B (**26**)–D (**28**) were isolated from *Punica granatum*. Tannins **27** and **28**, which both contain a gallagyl unit, were obtained together with punicalagin (**9**) from *Terminalia arborea* and *T. macroptera*, respectively.

The plant sources of *C*-glycosidic ellagitannins obtained from the order Myrtales are listed in Table 2.

### 2.3. Complex Tannins

Complex tannins (flavono-ellagitannins) are characterized by a unique C-C condensed structure of *C*-glycosidic tannins (vescalagin-type or stachyurin-type) with flavan-3-ol (catechin or epicatechin). Unlike the *C*-glycosidic tannins, these tannins have been found in a rather limited number of plant species belonging to the Combretaceae, Myrtaceae, Melastomataceae, Fagaceae, and Theaceae families [3].

**Figure 3.**
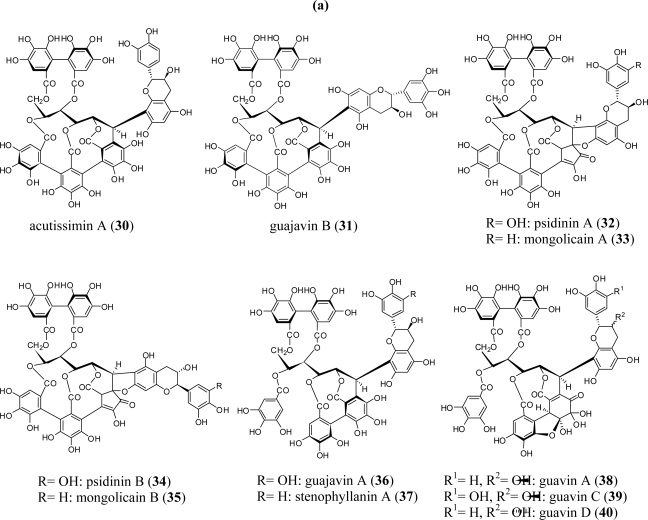

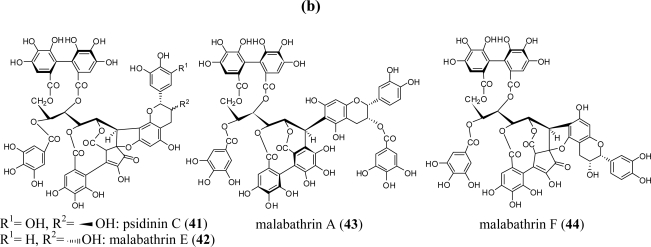
(a) Structures of complex tannins **30**–**40**. (b) Structures of complex tannins **41**–**44**.

A typical example of a vescalagin-based complex tannin, acutissimin A (**30**) was first isolated from fagaceous plants and later found in the combretaceous plant, *Anogeissus acuminata* var. *lanceolata* [43], and the myrtaceous plant, *Syzygium aqueum* [21]. Another myrtaceous plant, *Psidium guajava*, reportedly produces a diversity of complex tannins including **30** and its analogs guajavin B (**31**), psidinins A (**32**) and B (**34)**, and mongolicains A (**33**) and B (**35**); and the stachyurin-based analogs guajavin A (**36**), guavins A (**38**), C (**39**) and D (**40**), and psidinin C (**41**) [54] (Figure 3). *Melastoma malabathricum*, a member of the Melastomataceae, also produces metabolites from the stachyurin-based complex tannins malabathrins A (**43**), E (**42**), and F (**44**) [55].

A stachyurin-based congener, stenophyllanin A (**37**), was isolated from *Melaleuca squarrosa* (Myrtaceae) [52] and *Melastoma malabathricum* (Melastomataceae) [55].

It is noteworthy that both vescalagin- and stachyurin-based complex tannins hitherto isolated are all characterized by possessing a β-oriented C-C bond at glucose C-1 [1]. The formation of this class of tannins is rationalized by non-enzymatic diastereoselctive nucleophilic substitution reaction at the exo β-position of the benzylic C-1 cation where is less hindered than the α-site. In fact, many examples of hemisynthesis of the complex tannin by simple acid-catalized reaction between C-glycosidic tannin and (+)-catechin or (–)-epicatechin have been reported.

## 3. Oligomeric Ellagitannins

**Figure 4.**
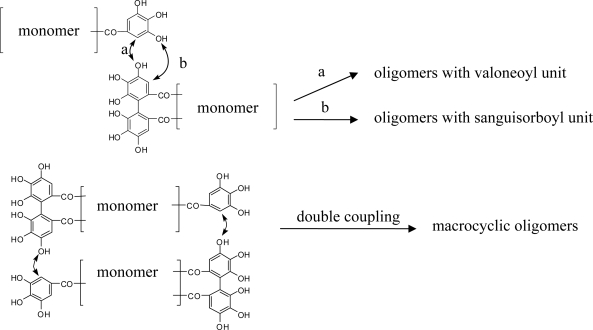
General oligomerization mode for the types 1 and 2. (1) examples of coupling mode for formation of valeoyl or its equivalent unit by C-O coupling. (2) macrocyclic dimer (double coupling for HHDP and galloyl).

Oligomeric ellagitannins are common among many plant families, including the Fagaceae, Rosaceae, Coriariaceae, Onagraceae, Melastomataceae, Myrtaceae, and Lythraceae [3]. This class of tannins is divided into three sub-groups based on structural features: (1) oligomers that contain a valoneoyl group or its equivalent, formed by intermolecular C-O bonds between an HHDP group and a galloyl group of a neighboring monomer, (2) macrocyclic oligomers formed by two C-O bonds, and (3) *C*-glycosidic tannin oligomers produced by intermolecular C-C bond formation between C-1 of one monomer and the aromatic ring of another (see Figure 4). These structural features are chemotaxonomically significant and are often characteristic of the plant genus or family. The following section provides an overview of the oligomers isolated thus far from each of the families within the Myrtales.

### 3.1. Oligomers from the Combretaceae

**Figure 5.**
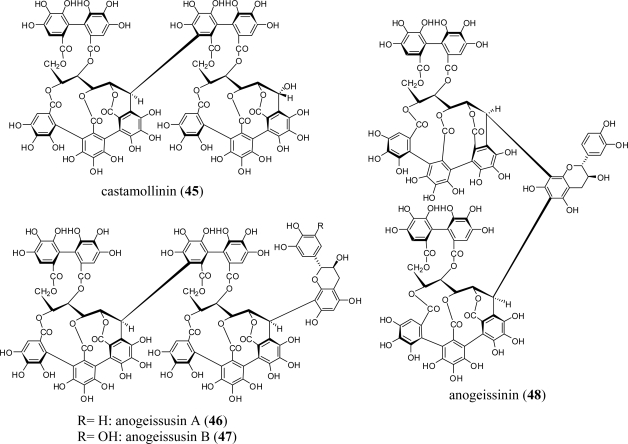
Structures of C-glycosidic ellagitannin dimers **45**–**48**.

Although more than 10 of the combretaceous plant species described above have yielded various ellagitannin monomers, only *Anogeissus acuminata* was reported to yield C-C linked dimers of *C*-glycosidic ellagitannin, including castamollinin (**45**), anogeissusins A (**46**) and B (**47**), and anogeissinin (**48**) [43] (Figure 5). Dimers **46**–**48** are relatively rare tannins in which two equivalents of vescalagin-type monomer are connected to or through the A-ring of a (+)-catechin or (+)-gallocatechin.

### 3.2. Oligomers from the Lythraceae and Onagraceae

**Figure 6.**
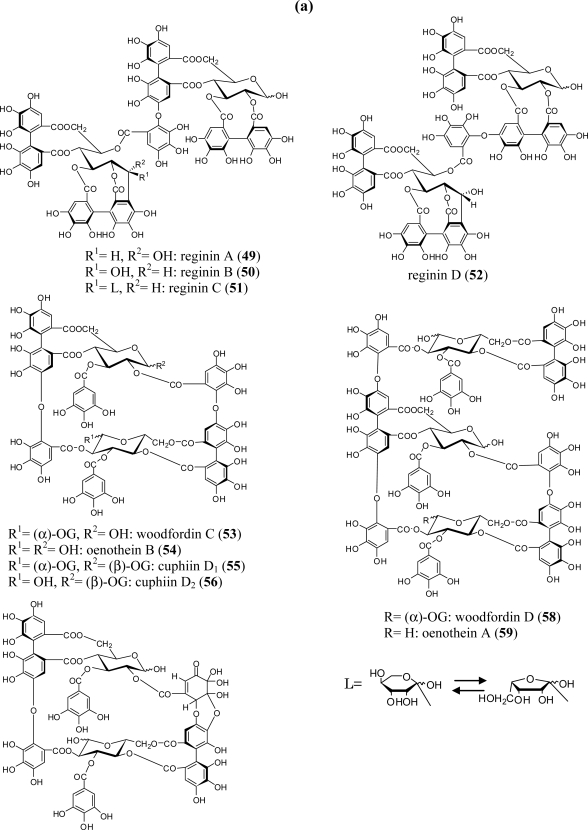

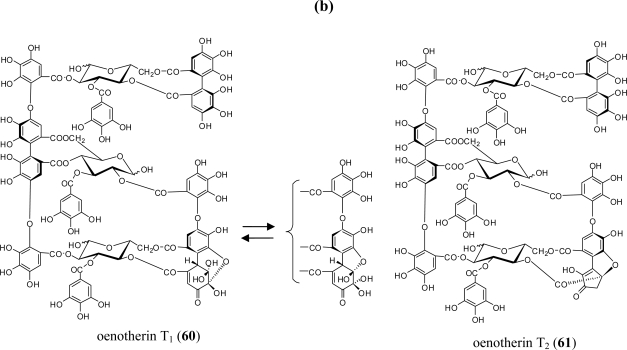
(a) Structures of ellagitannin oligomers **49**–**59**. (b) Structures of ellagitannin oligomers **60** and **61**.

The regio-isomeric dimers, reginins A (**49**) and D (**52**) together with reginins B (**50**) and C (**51**), which are produced by intermolecular C-O bonds between casuarinin (stachyurin) and pedunculagin, were isolated from *Lagerstroemia flos*-*reginea* (Lythraceae) [47] (Figure 6). Reginin A (**49**) has also been isolated from the leaves of *L. speciosa*, which are popular as “banaba” in the Philippines [48]. Unique macrocyclic oligomers, woodfordins C (**53**) and D (**58**) and their desgalloyl congeners oenotheins B (**54**) and A (**59**), were obtained from the leaves of *Woodfordia fruticosa*, one of the Jamu medicines in Indonesia [56]. Analogous macrocyclic dimers, cuphiins D_1_ (**55**) and D_2_ (**56**) co-occur with **53** and **54** in *Cuphea hyssopifolia,* a lythraceous shrub native to Mexico [57]. Oenotheins A (**59**) and B (**54**) were first isolated as the main tannins in *Oenothera erythrosepala* leaves [24] and are widely distributed in the *Oenothera* and *Epilobium* species of Onagraceae, *i.e.*, *O. laciniata* [25], *O. biennis* [58], *O. tetraptera* [26], *E. angustifolium* [23], and many other *Epilobium* species [59]. The occurrence of oxidized metabolites oenotherins T_1_ (**60**) and T_2_ (**61**) of **59** in *O. tetraptera* leaves was recently reported by Taniguchi *et al*. [26,60]. The chemical conversion of **60** to **59** was achieved by reduction with Na_2_S_2_O_4_.

### 3.3. Oligomers from Myrtaceae

**Figure 7.**
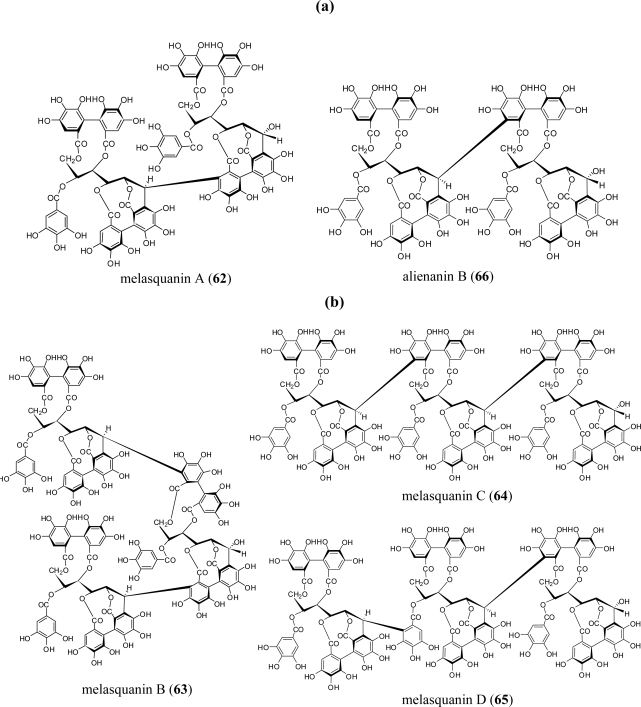
(a) Structures of ellagitannin oligomers **62** and **66**. (b) Structures of ellagitannin oligomers **63**–**65**.

**Figure 8.**
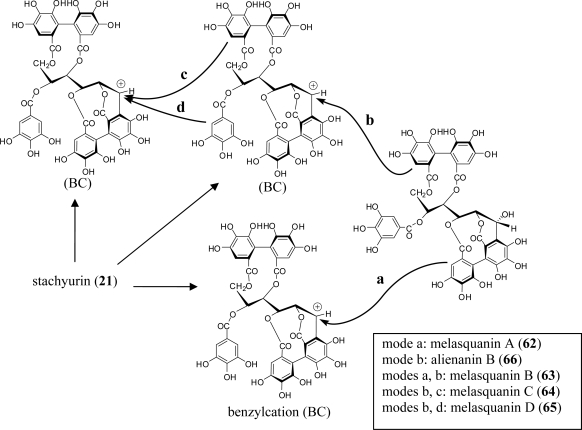
Coupling modes (a–d) to melasquanins A (**62**)–D (**65**).

**Figure 9.**
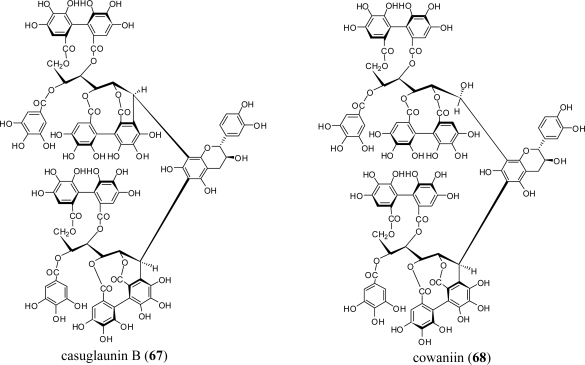
Structures of ellagitannin oligomers **67** and **68**.

In addition to the Lythraceae and Onagraceae, oenothein B (**54**) has been isolated from the myrtaceous plants *Eucalyptus alba* [15]*, Eucalyptus cypellocarpa* [61]*, Eucalyptus consideniana* [16], *Eugenia uniflora* [62], *Melaleuca leucadendron* [63], and *Myrtus communis* [19]. Of these plants, *E. uniflora*, *E. cypellocarpa,* and *M. communis* also produce eugeniflorin D_2_ (**57**) with a dehydrovaloneoyl group isomeric to that in oenotherin T_1_ (**60**). It recently has been shown that the leaves of *Melaleuca squarrosa,* an evergreen shrub indigenous to southeastern Australia, are rich in *C*-glycosidic ellagitannins including several new oligomers such as melasquanins A (**62**), B (**63**), C (**64**), and D (**65**), in addition to the previously reported alienanin B (**66**), and casuglaunins A and B (**67**) [52] (Figures 7–9). These oligomers may be biosynthesized through C-C bond formation facilitated by a nucleophillic attack (a–d) of the aromatic acyl ring of casuarinin (**20**) on β-site of the C-1 benzylic cation from stachyurin (**21**) (Figure 8) in a similar manner to that described in Sections 2–3.

The plant also yields a unique complex tannin dimer, cowaniin (**68**), first obtained from *Cowania mexicana* (Rosaceae) [64]. The chemical structure **68** inferred from spectral data was confirmed by conversion into **67** following an acid treatment.

### 3.4. Oligomers from Melastomataceae

**Figure 10.**
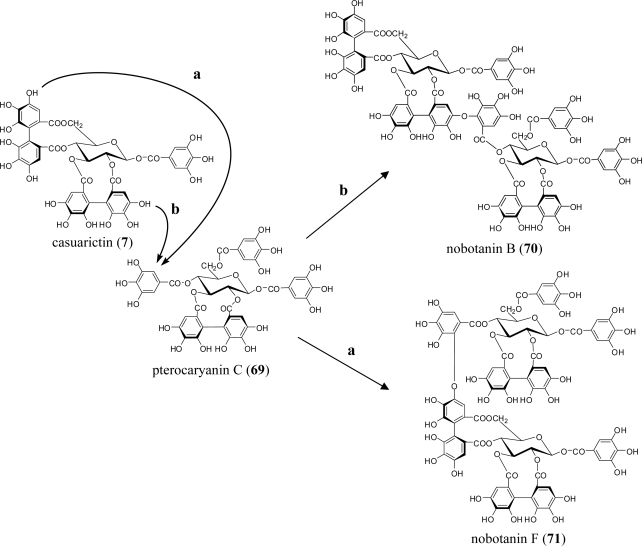
Coupling mode of nobotanins.

A series of studies on plant species in six genera (*Medinilla, Heterocentron, Tibouchina, Melastoma, Bredia,* and *Monochaetum*) of the Melastomataceae has revealed more than 20 characteristic ellagitannin oligomers up to pentamers, e.g., nobotanins A–C and E–T. These oligomers share two common features: (1) they are essentially composed of two different monomers, casuarictin (**7**; C) and pterocaryanin C (**69**; PC), which are coupled alternatively to form the valoneoyl unit; and (2) the galloyl group of **69** can only participate in the formation of the valoneoyl group at O-5, whereas the HHDP groups of both monomers are susceptible to bond formation regardless of their positions [65] (Figure 10).

**Figure 11.**
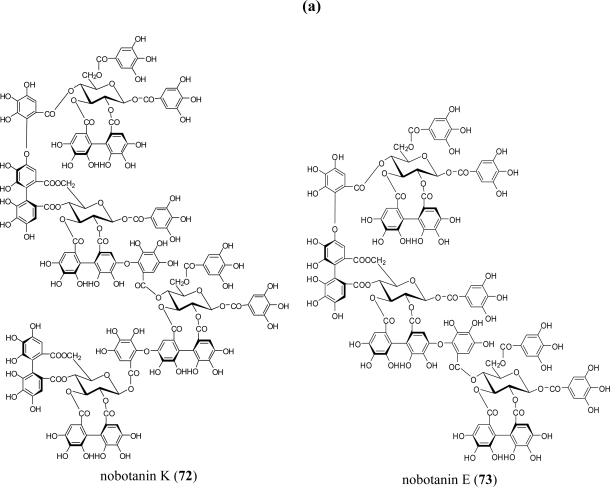

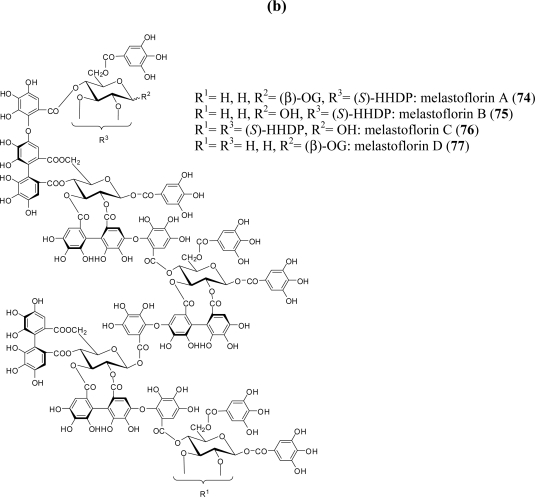
(a) Structures of ellagitannin oligomers **72** and **73**. (b) Structures of ellagitannin oligomers **74**–**77**.

These characteristics are chemotaxonomically significant relative to oligomers connected through the valoneoyl group, which are mostly constructed from a single monomeric component. The representative oligomers are nobotanins B (**70**; C-PC) [12], F (**71**; PC-C), and K (**72**; PC-C-PC-C), although **70** is the most abundant dimer in most species of this family. Nobotanin B (**70**) also seems to be a key compound from which trimers and tetramers are producible by further bonding with **7** and **69**, as observed in nobotanins E (**73**; PC-C-PC) and K (**72**) [66] (Figure 10). The largest pentameric oligomers, melastoflorins A (**74**) through D (**77**), were isolated together with several dimers and tetramers from the Colombian shrub *Monochaetum multiflorum* [65] (Figure 11).

## 4. Structure Determination of the Oligomeric Ellagitannins

**Figure 12.**
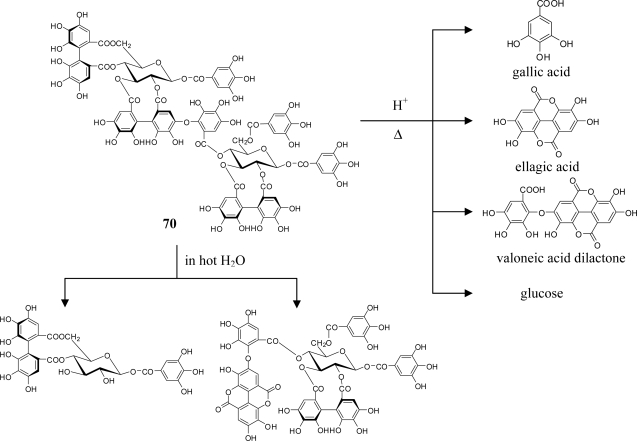
Chemical degradation of nobotanin B (**70**).

**Figure 13.**
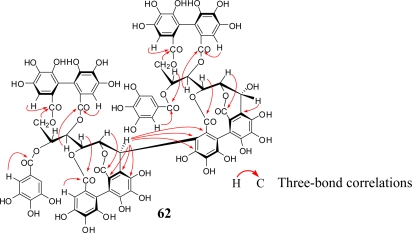
HMBC data for melasquanin A (**62**).

Structure elucidation of the oligomers has generally been achieved by (1) identification of their constituent units by methylation of the tannin followed by methanolysis or direct acid hydrolysis, (2) detailed spectroscopic analyses using MS, UV and NMR spectra including 2-dimensional ^1^H-^1^H (or ^1^H-^13^C) COSY and ^1^H-detected multi-bond heteronuclear multiple quantum coherence (HMBC), and (3) chemical confirmation of the structure presumed on the basis of the findings from the above (1) and (2) by the characterization of partial hydrolysates of smaller molecule in hot water as exemplified for nobotanin B (**70**) in Figure 12. Molecular weights up to 4,000 are nowadays determined with the aid of electrospray mass measurement in the presence of ammonium acetate, or FABMS ([M + H]^+^ or [M + Na]^+^). In the NMR analyses, HMBC provides a convenient and reliable way to determine the position of each acyl group on the glucose core by three-bond correlations between the aromatic proton and glucose proton through a common ester carbonyl carbon as illustrated for melasquanin A (**62**) in Figure 13. The atropisomerism of the chiral biphenyl moiety in the molecule is directly determined without any degradation reaction by circular dicroism (CD) spectrum in which positive or negative Cotton effect at around 230 nm is diagnostic for (*S*)- or (*R*)-configuration, respectively [8].

## 5. Biological Activities of Ellagitannins Found in the Myrtales

Remarkable progress in the structural characterization of the numerous tannins in foods, beverages, and medicinal plants since the 1980s has enabled *in vitro* and *in vivo* studies of their biological properties based on structural differences. A wide range of significant biological activities beneficial to human health have been reported for both ellagitannins and proanthocyanidins. The strong affinity of tannins to various biopolymers such as enzymes, and antioxidative effects based on radical scavenging, are key to their diverse biological effects [1]. A survey of the biological activity of the Myrtales tannins using the electronic search engines SciFinder Scholar and Science Direct revealed various antimicrobial, antitumor, enzyme-inhibitory, and immunomodulatory effects of ellagitannins encountered in species of Combretaceae, Lythraceae, Myrtaceae and Onagraceae, as shown in Table 3.

### 5.1. Casuarinin (**20**), Castalagin (**21**), and Related Tannins

Kolodziej *et al.* [69] evaluated the *in vitro* antileishmanial activity of various types of tannins using *Leishmania donovani*. Although none of the tannins showed significant antiparasitic effects against the extracellular promastigote of *L. donovani* (EC_50_ > 25 μg/mL), all of the hydrolysable tannins, including oligomers, exhibited potent activity (EC_50_ < 0.4–12.5 μg/mL) against the intracellular amastigote form which resides within murine macrophage-like RAW 264.7 cells infected with *L. donovani*. Observed potencies were stronger or comparable to that of the reference compound, Pentosam® (EC_50_ 7.9 μg/mL), which is therapeutically used as antileishmanial drug. Among the hydrolysable tannins, the most potent antileishmanial activity was exhibited by geraniin and related tannins (EC_50_ < 0.4 μg/mL). The *C*-glycosidic tannins casuarinin (**20**) and castalagin (**16**) also showed pronounced antileishmanial activities with EC_50_ values of 0.5 and 2.7 μg/mL, respectively. Note that most of these tannins, with the exception of oligomers, exhibited low cytotoxicity against murine host cells (EC_50_ > 25 μg/mL). Separate functional assays have shown that the amastigote-specific activity of these tannins is likely associated with immunomodulatory effects, such as macrophage activation to release cytokines, tumor necrosis factor (TNF)-α, and interferon (IFN)-γ. The degree of these immunomodulatory effects was highly correlated with the degree of intracellular *Leishmania* death. The search for antiparasitic substances in butanol extracts of *Anogeissus leiocarpus* and *Terminalia avicennoides,* which are used to treat some parasitic diseases in Africa, resulted in the characterization of castalagin (**16**) as a primary antileishmanial component with an EC_50_ ranging from 55 to greater than 150 μg/mL against the promastigote forms of four *Leishmania* strains [44].

Casuarinin (**20**) isolated from *Terminalia arjuna* also exhibits *in vitro* antivirus effects against Herpes simplex virus type 2 (HSV-2) with an IC_50_ of 3.6 and 1.5 μM in XTT and plaque reduction assays, respectively. These effects were associated with the inhibition of viral attachment and cell penetration [67]. Lin *et al.* [68] also found that **20** induced apoptosis in human breast adenocarcinoma MCF-7 cells and in human non-small cell lung cancer cells A549 by blocking cell cycle progression in the G0/G1 phase.

In the screening of spontaneously hypertensive rats, castalagin (**16**), chebulinic acid (**15**), and corilagin were identified as the major antihypertensive substances among the hydrolysable tannins isolated from the leaves of *Lumnitzera racemosa* (Combretaceae) [45].

Chebulagic acid (**14**) from *Terminalia chebula* has been shown to reversibly and non-competitively inhibit α-glucosidase (maltase) activity, suggesting a potential for managing type-2 diabetes [71]. Other tannins that have been identified as α-glucosidase inhibitors are tellimagrandin I (**1**) and eugeniin (casuarictin) (**7**) from *Syzygium aromaticum* (Myrtaceae) [70]. Recently, Reddy *et al.* reported that **14** also exhibited potent anti-inflammatory effects in mouse macrophage cell line RAW 264.7 that had been stimulated with LPS by inhibition of NF-κ.B activation and MAP kinase phosphorylation [73], and in COLO-205 cells by enzyme inhibition of COX and 5-LOX [72].

### 5.2. Punicalagin **(9)** and Related Tannins

Hepatoprotective effects of various tannins based on their ability to scavenge radical reactive oxygen species (ROS) have been demonstrated both *in vitro* and *in vivo*. For example, punicalagin (**9**) and punicalin (**10**) from *Terminalia* species exhibited inhibitory effects on hepatotoxicity induced by acetaminophen [75] and CCl_4_ [38]. Other activities associated with the antioxidative effects of punicalagin (**9**) include the suppression of bleomycin-induced genotoxicity in cultured Chinese hamster ovary cells [76] and of the proliferation of H-ras-transformed NIH3T3 cells. These effects are due, in part, to decreases in intracellular superoxide levels, which may modulate downstream signaling of Ras protein [77].

### 5.3. Lagerstroemin (**29**)

*Lagerstroemia speciosa* (Lythraceae) has been used as an herbal medicine for the treatment of diabetes in the Philippines. Screening of the plant extract identified lagerstroemin (**29**), flosin B (C_1_-epimer of **29**), and reginin A (**49**) as activators of glucose transport using rat fat cells, all of which are characteristic *C*-glycosidic ellagitannins of the plant [78]. The insulin-like activity of **29** was indicated by increases in glucose uptake by rat adipocytes, and by increased tyrosine-phosphorylation in Chinese hamster ovary cells expressing human insulin receptors [79]. In addition, casuarinin (**20**), stachyurin (**21**), and casuariin (**22**) as well as **29** were identified as active components in the stimulation of insulin-like glucose uptake and in the inhibition of adipocyte differentiation (**20** and **29**) in 3T3-L1 cells [80].

### 5.4. Oenothein B (**54**) and Related Macrocyclic Oligomers

Macrocyclic oenothin B (**54**) reportedly exhibited remarkable host-mediated antitumor activity with intraperitoneal injection several days before inoculation of sarcoma 180 tumor cells into the abdomen of mice [24]. Evaluation of activity was gauged by the number of survivors and the percent increase in life span (%ILS) 60 days after administration. Treatment with a 10 mg/kg dose of oenothein B (**54**) resulted in 4 survivors out of 6 mice and 196% ILS, the most potent results of among the approximately 100 polyphenols evaluated. This activity was related to an immunomodulatory effect consisting of macrophage activation and consequent release of cytokine interleukin-1β [87]. Woodfordin C (**53**) also exhibited a potent activity with 160% ILS and one survivor out of five mice after 60 days [56]. The potent activity of the oligomeric ellagitannins stands in contrast to the negligible activity observed with most of the monomeric hydrolysable tannins, proanthocyanidins, and related low-molecular weight polyphenols.

Woodfruticosin (woodfordin C) (**53**) was also an effective inhibitor (IC_50_ 2.5 μg/mL) of deoxyribonucleic acid topoisomerase II, the potency of which was 10-fold stronger than that of adriamycin and etoposide in molar concentrations [81].

Eugeniflorin D_1_ and D_2_ (**57**) as well as oenothein B (**54**) obtained from the extract of *Eugenia uniflora* (Myrtaceae) were efficient inhibitors of Epstein-Barr virus (EBV) DNA polymerase, a key enzyme for replication of EBV associated with nasopharyngeal carcinoma [82].

Using activity-guided fractionation for bioactive components of *Epilobium* species, Ducrey *et al*. [59] showed that oenothein A (**59**) and B (**54**) are potent inhibitors of 5α-reductase and aromatase, which are involved in the etiology of benign prostatic hyperplasia.

Biological studies of an oenothein B analog, cuphiin D_1_ (**55**), isolated from *Cuphea hyssopifolia* (Lythraceae) revealed antitumor effects through the induction of apoptosis in human promyelocytic leukemia (HL-60) cells and human cervical carcinoma (HeLa) cells [85]. Cuphiin D_1_ (**55**) was also shown to activate human peripheral blood mononuclear cells to release cytokines IL-1β, IL-2 and TNF-α [84].

Many pathogenic bacteria, such as methicillin-resistant *Staphylococcus aureus* (MRSA), have acquired resistance to various clinical antibiotics. This worldwide problem is driving the development of new antibiotic drugs. Observed synergistic effects of certain polyphenols such as oenothein B (**54**) and tellimagrandin I (**1**) have been suggested as a means to restore the effectiveness of β-lactam antibiotics against MRSA. When used together with these tannins, the MICs of oxacillin against MRSA strains were markedly lowered to 1/250 or 1/500 [88]. These results may represent one strategy for overcoming emergent bacterial resistance.

### 5.5. Nobotanins

In a survey for new, natural anticancer chemotherapeutic drugs, some oligomeric ellagitannins showed promise as inhibitors of poly(ADP-ribose) glycohydrolase, which is associated with gene activation upon DNA repair, replication, and transcription [86]. During initiation of gene expression, DNA replication, and cell differentiation, poly(ADP-ribose) from specific chromosomal proteins is degraded primarily by poly(ADP-ribose) glycohydrolase to yield ADP-ribose and mono(ADP-ribosyl) proteins. It has been suggested that this degradation of poly(ADP-ribose) is an important factor in the regulation of gene activation. Ellagitannins showed an appreciable inhibitory effect with an IC_50_ of 0.3–11.9 μM on poly(ADP-ribose) glycohydrolase purified from human placenta. Procyanidin oligomers and their constituent flavan-3-ols were inactive even at concentrations of 100 μM. Potent activity was exhibited by oligomeric ellagitannins, including dimers such as oenothein B (**54**) (IC_50_ 4.8 μM) and nobotanin B (**70**) (IC_50_ 4.4 μM), a trimer (nobotanin E (**73**), IC_50_ 1.8 μM), and a tetramer (nobotanin K (**72**), IC_50_ 0.3 μM).

## 6. Conclusions

A large number of ellagitannins have been isolated and characterized from a wide array of plant sources during the last several decades. The plants from which individual ellagitannins were first isolated belonged largely to the order Myrtales. Most notably, several *Terminalia* species of Combretaceae produce punicalagin and its congeners, all of which contain a unique gallagyl group, previously found only in *Punica granatum* (Punicacease). These findings imply a close chemotaxonomic relationship between these plants. Approximately 40% of the oligomeric ellagitannins characterized thus far were initially isolated from species of Onagraceae, Lythraceae, Myrtaceae, Trapaceae, and Melastomataceae, indicating that these plant varieties are good natural sources of these oligomers. In particular, macrocyclic tannins, which include oenothein B and its analogs, are characteristic of the Onagraceae, Lythraceae, and Myrtaceae. Various *in vitro* and *in vivo* assays have demonstrated diverse biological activities for these ellagitannins and indicate the potential of these materials as antioxidant food additives [89]. However, although there are several reports that identify ellagitannin metabolites in animal urine and feces, e.g., ellagic acid derivatives (**77**, **78**) [90] and compounds **79**–**84** [91], the bioavailability of these tannins in humans has not been studied extensively.

Further studies in this field will include characterization of immunomodulating effects in the digestive tract that could clarify the role(s) of ellagitannins in human health and help explain their widespread use in traditional medicines.

## Figures and Tables

**Figure 14.**
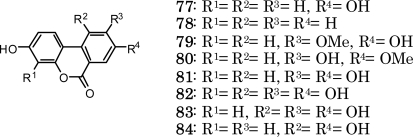
Structures of metabolites from ellagitannins.

**Table 1.** Ellagitannin monomers found in the Myrtales.

**Tannin**	**1**	**2**	**3**	**4**	**5**	**6**	**7**	**8**	**9**	**10**	**11**	**12**	**13**	**14**	**15**
**Plant source [Ref]**															
Trapaceae															
*Trapa japonica* [9]	+			+		+	+								
Melastomataceae															
*Bredia tuberculata* [10]						+	+								
*Heterocentron roseum* [11]			+				+								
*Melastoma malabathricum* [12]			+			+	+								
*M. normale* [10]			+			+	+								
*Tibouchina semidecandra* [13]						+	+	+							
Myrtaceae															
*Callistemon lanceolatus* [14]				+		+		+							
*Eucalyptus alba* [15]	+			+		+		+							
*E. consideniana* [16]	+		+	+		+									
*E. globulus* [17]	+														
*E. rostrata* [18]	+														
*E. viminalis* [16]	+	+		+		+									
*Myrtus communis* [19]	+	+													
*Pimenta dioica* [20]		+	+		+	+									
*Syzygium aqueum* [21]		+				+	+								
*S. aromaticum* [22]	+	+	+	+			+								
Onagraceae															
*Epilobium angustifolium* [23]	+		+	+		+									
*Oenothera erythrosepala* [24]	+			+											
*O. laciniata* [25]	+														
*O. tetraptera* [26]	+	+		+											
Combretaceae															
*Combretum glutinosum* [27]								+	+	+					
*C. molle* [28]									+	+					
*Quisqualis indica* [29]	+	+				+		+	+	+					
*Terminalia arborea* [30]								+	+	+				+	+
*T. arjuna* [31]								+	+	+					
*T. brachystemma* [32]									+						
*T. calamansanai* [33]	+	+						+	+	+					
*T. catappa* [34]	+							+	+	+	+	+	+	+	+
*T. chebula* [35]									+	+		+	+	+	+
*T. citrina* [36]									+					+	
*T. macroptera* [37]								+	+			+	+		
*T. myriocarpa* [38]								+	+						
*T. triflora* [39]										+					
Punicaceae															
*Punica granatum* [40]	+		+			+		+	+	+					

**Table 2.** *C*-Glycosidic ellagitannins in the order Myrtales.

**Family**	**Plant species**	***C*-Glycosidic tannins**	**Ref.**
Combretaceae	*Anogeissus acuminata*	**16**, **17**, **18**, **19**	[43]
	*Anogeissus leiocarpus*	**16**	[44]
	*Lumnitzera racemosa*	**16**	[45]
	*Terminalia arjuna*	**16**, **20**, **22**	[31]
	*Terminalia macroptera*	**27**	[37]
	*Terminalia arborea*	**28**	[30]
	*Thiloa glaucocarpa*	**16**, **18**, **20**, **21**	[46]
Lythraceae	*Lagerstroemia flos-reginea*	**16**, **18**, **20**, **21**, **22**, **23**, **24**, **29**	[47]
	*Lagerstroemia speciosa*	**16**, **18**, **19**, **29**	[48]
Melastomataceae	*Osbeckia chinensis*	**20**, **22**, **25**	[49]
	*Tibouchina semidecandra*	**16**, **18**, **20**	[13]
Myrtaceae	*Callistemon lanceolatus*	**20**	[14]
	*Eucalyptus alba*	**21**, **22**	[15]
	*Eugenia grandis*	**16**, **18**	[50]
	*Kunzea ambigua*	**20**	[51]
	*Melaleuca squarrosa*	**20**, **21**	[52]
	*Pimenta dioica*	**16**, **18**, **20**, **22**	[20]
	*Siphoneugena densiflora*	**16**, **20**	[53]
	*Syzygium aqueum*	**16**, **18**, **19**	[21]
	*Syzygium aromaticum*	**20**, **22**	[22]
Punicaceae	*Punica granatum*	**20**, **22**, **25**, **26**, **27**, **28**	[40]
Trapaceae	*Trapa japonica*	**20**	[9]

**Table 3.** Biological activities of ellagitannins found in the Myrtales.

**Biological activity**	**Compound (source)**	**Ref.**
Anti-Herpes simplex virus type 2 activity	casuarinin (**20**) (*Terminalia arjuna*)	[67]
Apoptosis in human breast adenocarcinoma MCF-7 cells	casuarinin (**20**)	[68]
Antileishmanial activity	casuarinin (**20**), castalagin (**16**)	[69]
	castalagin (**16**) (*Anogeissus leiocarpus*)	[44]
Antihypertensive activity (rats)	castalagin (**16**) (*Lumnitzera racemosa*)	[45]
	corilagin, chebulinic acid (**15**)	
α-Glucosidase inhibitor	casuarictin (**7**) (*Syzygium aromaticum*)	[70]
	chebulagic acid (**14**) (*Terminalia chebula*)	[71]
Dual inhibitor against COX and 5-LOX	chebulagic acid (**14**) (*T. chebula*)	[72]
Anti-inflammation in LPS-induced RAW 264.7 cells	chebulagic acid (**14**) (*T. chebula*)	[73]
Effect on carageenan-induced inflammation	punicalagin (**9**), punicalin (**10**) (*T. catappa*)	[74]
Antioxidant and hepatoprotective effects on acetaminophen-induced liver damage in rats	punicalagin (**9**), punicalin (**10**) (*T. catappa*)	[75]
Effect against bleomycin-induced genotoxicity in Chinese hamster ovary cells	punicalagin (**9**) (*T. catappa*)	[76]
Chemopreventive effect on H-ras-transformed NIH3T3 cells	punicalagin (**9**) (*T. catappa*)	[77]
Inhibitory effect on HIV-1 reverse transcriptase	punicalin (**10**), 2-*O*-galloylpunicalin (*T. triflora*)	[39]
Inhibitory effect on CCl_4_-induced hepatotoxicity	punicalagin (**9**) (*T. myriocarpa*)	[38]
Activators of glucose transport in fat cells	lagerstroemin (**29**), reginin A (**49**) (*L. speciosa*)	[78]
Activation of insulin receptors	lagerstroemin (**29**)	[79]
Insulin-like glucose uptake-stimulatory/inhibitory and adiposities differentiation inhibitory activity in 3T3-L1 cells	lagerstroemin (**29**) casuarinin (**20**), casuariin (**22**), stachyurin (**21**)	[80]
Host-mediated antitumor effect	oenothein B (**54**) (*Oenothera erythrosepala*)	[24]
Host-mediated antitumor	oenothein B (**54**) (*Woodfordia fruticosa*)	[56]
	woodfordins A-C (**53**)	
Inhibitor of deoxyribonucleic acid topoisomerase II	woodfruticosin [= woodfordin C (**53**)]	[81]
EBV DNA polymerase inhibitory effect	oenothein B (**54**) (*Eugenia uniflora*)	[82]
	eugeniflorins D_1_, D_2_ (**57**)	
5α-reductase, aromatase inhibitory effect	oenotheins A (**59**), B (**54**) (*Epilobium* sp)	[59]
Induction of neutral endopeptidase activity in PC-3 cells	oenothein B (**54**) (*Epilobium angustifolium*)	[83]
*In vitro* immunomodulatory effect on human mononuclear cells	cuphiin D_1_ (**55**) (*Cuphea* sp)	[84]
Induce apoptosis in HL-60 cells	cuphiin D_1_ (**55**)	[85]
Poly (ADP-ribose) glycohydrolase inhibition	nobotanins B (**70**), K (**72**) (*Tibouchina* sp)	[86]

L. speciosa: Lagerstroemia speciosa.
